# Prevalence of Electronic Cigarette Dependence Among Youth and Its Association With Future Use

**DOI:** 10.1001/jamanetworkopen.2019.21513

**Published:** 2020-02-05

**Authors:** Erin A. Vogel, Junhan Cho, Rob S. McConnell, Jessica L. Barrington-Trimis, Adam M. Leventhal

**Affiliations:** Stanford Prevention Research Center, Department of Medicine, Stanford University, Stanford, California; Keck School of Medicine, Department of Preventive Medicine, University of Southern California, Los Angeles; Keck School of Medicine, Department of Preventive Medicine, University of Southern California, Los Angeles; Keck School of Medicine, Department of Preventive Medicine, University of Southern California, Los Angeles; Keck School of Medicine, Department of Preventive Medicine, University of Southern California, Los Angeles; Department of Psychology, University of Southern California, Los Angeles

## Abstract

**IMPORTANCE:**

Understanding the prevalence and symptoms of electronic cigarette (e-cigarette) dependence and its association with future e-cigarette use among youth may help to guide pediatric clinical services and health policy.

**OBJECTIVES:**

To examine the cross-sectional prevalence and symptom presentation of e-cigarette dependence and to determine whether e-cigarette dependence is associated with subsequent e-cigarette use patterns 6 months later among youth with baseline past-year e-cigarette use.

**DESIGN, SETTING, AND PARTICIPANTS:**

This prospective cohort study used baseline and 6-month follow-up surveys among students in the 12th grade during the 2016 to 2017 school year who reported any past-year e-cigarette use. Surveys were conducted on site in 10 high schools in Los Angeles, California. Data were analyzed from March 2019 to December 2019.

**MAIN OUTCOMES AND MEASURES:**

Self-reported checklist of 10 tobacco product dependence symptoms reflecting loss of control over use, craving or urge, or withdrawal symptoms while abstinent, completed at baseline and administered separately for e-cigarettes and combustible cigarettes. Reporting 1 or more symptoms indicated a positive screen for dependence. Vaping continuation, defined as any past 6-month vaping, and past 30-day nicotine vaping days (range, 0–30), sessions per vaping day (range, 0–20), and puffs per session (range, 0–20) at 6-month follow-up were assessed.

**RESULTS:**

Among 3168 twelfth-grade students who completed the baseline survey, 444 youths (mean [SD] age, 17.48 [0.39] years; 217 [48.9%] female) reported past-year e-cigarette use. Among these, 52 youths (11.7%) reported at least 1 e-cigarette dependence symptom. Among youth who reported past-year dual e-cigarette and combustible cigarette use, combustible cigarette dependence, reported by 43 youths (29.7%), was more prevalent than e-cigarette dependence, which was reported by 24 youths (16.4%). The most common symptoms, craving, urge, and need to use, and least common symptoms, abstinence-related concentration and emotional problems, were similar in both combustible and e-cigarette dependence. The prevalence of e-cigarette dependence was higher among youth who reported vaping in the past month than among those who did not (41 youths [17.6%] vs 11 youths [5.2%]; *P* < .001) and among youth who used e-cigarettes with nicotine than among those who used e-cigarettes without nicotine (42 youths [15.2%] vs 10 youths [6.0%]; *P* = .004). After adjusting for baseline vaping and e-cigarette dependence risk propensity scores, baseline e-cigarette dependence symptom status was associated with vaping continuation (adjusted odds ratio, 2.30 [95% CI, 1.07–4.94]; *P* = .02) and past 30-day number of nicotine vaping days (adjusted rate ratio, 2.17 [95% CI, 1.44–3.28]; *P* < .001), vaping sessions per day (adjusted rate ratio, 2.41 [95% CI, 1.52–3.83]; *P* < .001), and puffs per session (adjusted rate ratio, 1.70 [95% CI, 1.09–2.66]; *P* = .02) at 6-month follow-up.

**CONCLUSIONS AND RELEVANCE:**

These findings suggest that e-cigarette dependence may be an expression of tobacco use disorder associated with future use persistence and escalation among youth. Electronic cigarette dependence may be a behavioral health consequence of adolescent vaping that warrants consideration in pediatric patient care and public health policy.

## Introduction

Tobacco dependence is particularly concerning in adolescence, when the developing brain is especially vulnerable and dependence symptoms may arise after minimal exposure.^1,2^ Presently, electronic cigarettes (e-cigarettes), which provide pulmonary nicotine and therefore possess high dependence potential,^3,4^ are the most popular tobacco product among US youth.^5^ The occurrence of e-cigarette dependence symptoms and their association with nicotine exposure have been documented^6–9^; however, foundational evidence on the symptom presentation, prevalence, and subgroups at elevated risk of e-cigarette dependence among youth is lacking, as is information on its association with future e-cigarette use, to our knowledge.

The objective of this prospective cohort study was to examine the prevalence and symptom expression of e-cigarette dependence and its association with future e-cigarette use among past-year e-cigarette users aged 16 to 18 years in Southern California. Specifically, we examined similarities and differences in prevalence and symptom expression between e-cigarette dependence and combustible cigarette dependence among youth, the prevalence of e-cigarette dependence symptoms stratified by subgroups presumed to be at elevated risk (ie, youth who vaped e-cigarettes with nicotine, vaped in the past month, or were dual users of e-cigarettes and combustible cigarettes), and associations of baseline e-cigarette dependence with subsequent vaping continuation, frequency, and intensity patterns 6 months later. This study aims to provide foundational descriptive evidence on the expression and progression of e-cigarette dependence as a potential presentation of tobacco use disorder among youth. Such evidence could help establish whether e-cigarette dependence is a health outcome of e-cigarette use that should be considered in federal regulatory decisions that weigh the relative harms and benefits of e-cigarettes.

## Methods

### Participants and Procedures

Data were drawn from the Happiness & Health Study,^10^ a prospective cohort study of behavioral health. All ninth-grade students in 10 participating public high schools in Los Angeles County, California, in 2013 were eligible. Semiannual in-classroom assessments were administered from 2013 to 2017. Students who were not in class on survey days completed abbreviated surveys, which excluded dependence measures. Data on e-cigarette dependence were first collected in the fall 12th-grade survey in 2016, which was considered baseline; follow-up data were collected in the spring of 12th grade, approximately 6 months later, in 2017.

The University of Southern California Institutional Review Board approved this study. Participants provided active assent and a parent or legal guardian provided written or verbal informed consent prior to study enrollment. This study is reported according to Strengthening the Reporting of Observational Studies in Epidemiology (STROBE) reporting guideline.

### Measures

#### e-Cigarette and Combustible Cigarette Dependence Symptoms

At baseline, tobacco product dependence symptoms were measured using the Hooked on Nicotine Checklist,^11^ which was originally developed to measure combustible cigarette dependence and has demonstrated adequate psychometric properties. Students reported whether they had ever experienced each of 10 dependence symptoms for e-cigarettes and combustible cigarettes separately. Electronic cigarette and combustible cigarette dependence items were identically worded, except for substitution of *e-cigarette* and *vaping* terms for *cigarette* and *smoking*. Endorsing 1 or more symptoms indicated that the participant screened positive for dependence.^11^ Students presenting 2 or more or 3 or more total symptoms of e-cigarette or combustible cigarette dependence symptoms were also classified.

#### e-Cigarette and Other Tobacco Product Use

At baseline, use of e-cigarettes with nicotine and e-cigarettes without nicotine or cannabis oil were measured as yes-or-no questions.^12^ Affirmative responses to either or both questions over the past year were used to classify past-year any e-cigarette use (yes or no), which was necessary for sample inclusion. Past-month vaping (yes or no) and past-year vaping of e-cigarettes that contained nicotine (yes or no) were also assessed. We assessed baseline past-year combustible cigarette smoking (yes or no) using items from previously derived epidemiologic surveys.^12^

Vaping continuation was operationalized as any past 6-month use of e-cigarettes (with or without nicotine) at follow-up. Additional survey items assessed past 30-day nicotine vaping frequency (range, 0–30 days) and intensity, including number of nicotine vaping sessions per vaping day (range, 0–20) and puffs per nicotine vaping session (range, 0–20) (eAppendix in the Supplement).^13^

#### Covariates

We collected several additional measures to describe the sample and assess risk factors for e-cigarette dependence to be included in an e-cigarette propensity score covariate. These variables may have also influenced e-cigarette use progression patterns and therefore confounded associations between e-cigarette dependence and future use.

Participants reported age (in years) at e-cigarette use initiation. The following tobacco product use characteristics were measured: past 30-day number of days smoked cigarettes (range, 0–30), cigarettes smoked per day on smoking days (range, 0–20), and ever (lifetime) use of cigars, hookah, or smokeless tobacco (yes or no).^14^ Participants reported ever use of alcohol, combustible cannabis, vaporized cannabis, or other drugs (yes or no) using questions derived from previously validated items.^15^

To assess a potential association of mental health with e-cigarette dependence and future use, we measured symptoms of major depressive disorder, generalized anxiety disorder, social phobia, panic disorder, obsessive-compulsive disorder, manic symptoms, attention-deficit/hyperactivity disorder, and conduct disorder symptoms (eAppendix in the Supplement).^16–19^ Additionally, age, sex (male or female), self-reported race/ethnicity (ie, Hispanic, white, Asian, black, or other) and highest level of parental education (ie, college degree or greater or less education) were surveyed, per past work.^10^

#### Statistical Analysis

After descriptive analyses, we reported prevalence of e-cigarette dependence (report of ≥1 symptom) and prevalence of the 10 specific dependence symptoms among all past-year e-cigarette users and by combustible cigarette use status. Among past-year e-cigarette and combustible cigarette dual users, McNemar tests for within-participant comparisons were used to conduct cross–tobacco product comparisons of e-cigarette dependence, combustible cigarette dependence, and individual symptoms. Prevalence of e-cigarette dependence symptoms was compared across binary subclassifications of past-year vaping of e-cigarettes with (vs without) nicotine, past-month vaping (yes or no), and past-year combustible cigarette and e-cigarette dual use (vs e-cigarette use only) using χ^2^ tests. For descriptive data, prevalence of meeting 2 or more (yes or no) or 3 or more (yes or no) symptom thresholds were also reported. The prospective association of baseline e-cigarette dependence symptom status with nicotine vaping status at follow-up was tested using binary logistic regression. Prospective associations of baseline e-cigarette dependence symptom status with nicotine vaping frequency and intensity at follow-up were tested using negative binomial regression models. All regressions included baseline status of each respective outcome as a covariate. Adjusted analyses included an e-cigarette dependence propensity score covariate calculated from a prediction model of e-cigarette dependence status regressed on 25 baseline variables, as detailed in the eAppendix in the Supplement. Additional and supplemental sensitivity analyses were conducted. Analyses were tested in Mplus statistical software version 7 (Muthen & Muthen) using full information maximum likelihood estimating to account for missing data, and participants’ high school (clustering by school) was accounted for using complex modeling. For primary analyses, Benjamini-Hochberg multiple-testing corrections^20^ were applied to control studywise false-discovery rate at 0.05, based on 2-tailed corrected *P* values. Data were analyzed from March 2019 to December 2019.

## Results

### Study Sample

**Figure 1.**
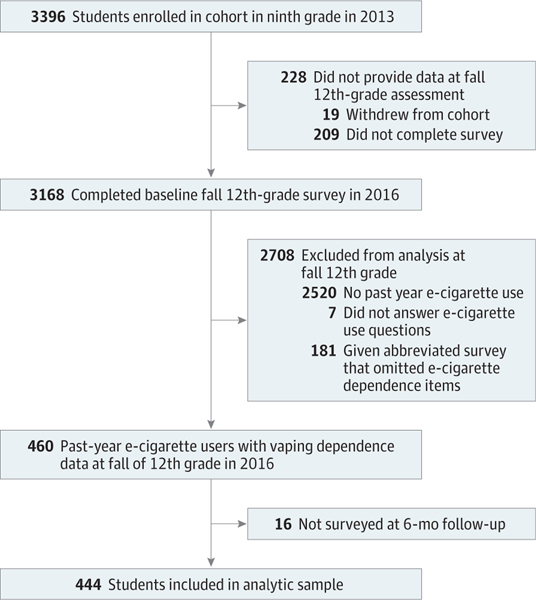
Study Accrual Flowchart e-Cigarette indicates electronic cigarette.

Among 4100 eligible students enrolled in ninth grade in the Fall 2013 semester, 3396 students enrolled in the cohort, and 3168 students completed the fall 12th-grade baseline survey in 2016. Of these students, 460 reported past-year use of e-cigarettes with or without nicotine and had baseline data on key measures. Sixteen students lacked follow-up data and were excluded from the analytic sample (Figure 1). The final sample included 444 youths (mean [SD] age, 17.48 [0.39] years; 217 [48.9%] female) (Table 1). There were no significant differences in mean age or parental education levels across the analytic sample compared with those who were lost to follow-up or who were not in class and received the abbreviated survey (eTable 1 in the Supplement).

### Cross-Sectional Analyses

#### Sample Characteristics

Among 444 youths who reported past-year e-cigarette use, the mean (SD) age of vaping initiation was 15.31 (1.58) years (Table 1). Of past-year users, 276 youths (62.2%) used e-cigarettes with nicotine, 233 youths (52.5%) vaped in the past month, and 146 youths (32.9%) reported past-year dual use of e-cigarettes and combustible cigarettes. Most (287 youths [65.2%]) had also used other tobacco products (eg, hookah, cigars) or other substances, including cannabis (340 youths [77.8%]) or alcohol (402 youths [91.8%]). Full distributions of tobacco product use characteristics are reported eTable 2 in the Supplement. Comparisons of study covariates by e-cigarette dependence symptom status showed that youth who had 1 or more e-cigarette dependence symptoms, compared with those who had none, vaped nicotine at higher frequency (mean [SD] vaping days in past 30 days, 6.10 [10.85] vs 1.17 [4.35]; *P* < .001) and intensity (mean [SD] sessions per vaping day, 5.25 [7.42] vs 1.05 [3.60]; *P* < .001), were more likely to report use of other tobacco products (43 youths [84.3%] vs 244 youths [62.7%]; *P* = .002), smoke more combustible cigarettes per day (mean [SD] cigarettes per day, 1.29 [3.81] vs 0.39 [1.85]; *P* = .005), vape cannabis (32 youths [62.7%] vs 180 youths [46.4%]; *P* = .04), and experience higher mean (SD) symptom levels of major depressive disorder (11.92 [8.03] vs 7.65 [6.97]; *P* < .001), mania (7.04 [3.61] vs 5.23 [3.64]; *P* = .001), generalized anxiety disorder (8.20 [4.60] vs 6.74 [4.70]; *P* = .04), panic disorder (5.63 [6.28] vs 4.05 [5.14]; *P* = .04), and conduct problems (18.34 [9.85] vs 15.60 [6.34]; *P* = .008) (Table 1). Descriptive comparisons by nicotine use status found that participants who used e-cigarettes with nicotine in the past year, compared with those who used e-cigarettes without nicotine, were more likely to report any past 6-month e-cigarette use (140 youths [50.7%] vs 44 youths [26.2%]; *P* < .001) and reported higher mean (SD) past-month number of nicotine vaping days (4.01 [8.34] vs 0.38 [1.92]; *P* < .001), nicotine vaping sessions (2.59 [5.66] vs 0.20 [0.91]; *P* < .001), and puffs per nicotine session (1.84 [3.97] vs 0.38 [1.30]; *P* < .001) (eTable 2 in the Supplement).

#### e-Cigarette Dependence in Overall Sample

**Figure 2.**
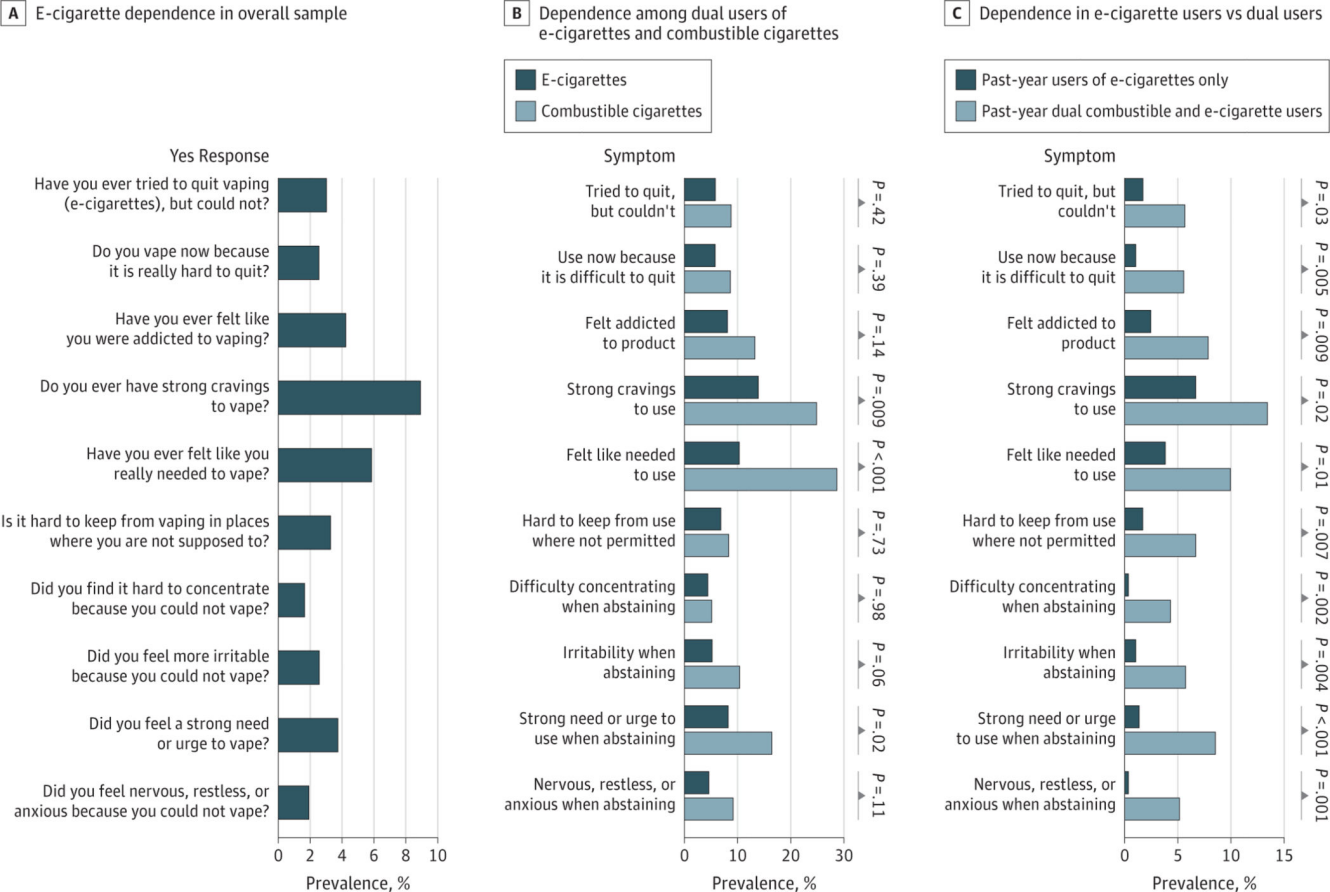
Electronic Cigarette (e-Cigarette) and Combustible Cigarette Dependence Prevalence and Symptom Profiles

The profile of prevalence rates for each of the 10 different tobacco product dependence symptoms for e-cigarettes and combustible cigarettes are reported in Figure 2. Among all past-year users of e-cigarettes with or without nicotine, 52 youths (11.7%) reported at least 1 e-cigarette dependence symptom at baseline; while 34 youths (7.7%) reported at least 2 e-cigarette dependence symptoms, and 22 youths (5.0%) reported at least 3 e-cigarette dependence symptoms. eTable 3 in the Supplement presents detailed frequencies across the distribution of 0 to 10 total symptoms. In all past-year e-cigarette users, the most commonly reported symptoms were experiencing strong urges to use (38 youths [8.9%]), feeling the need to use (25 youths [5.8%]), and feeling addicted to e-cigarettes (18 youths [4.2%]) (Figure 2A). The least common symptoms were difficulty concentrating when abstaining (7 youths [1.6%]), nervousness, restlessness, or anxiousness when abstaining (8 youths [1.9%]), using because it is difficult to quit (11 youths [2.5%]), and irritability when abstaining (11 youths [2.6%]).

#### Cross-Product Comparison in Dual Users of Combustible and e-Cigarettes

Among 146 baseline past-year dual users of e-cigarettes and combustible cigarettes, youth were nearly 2-fold as likely to report at least 1 combustible cigarette dependence symptom than e-cigarette dependence symptom (43 youths [29.7%] vs 24 youths [16.4%]; *P* < .001) and were more likely to meet 2 or more (40 youths [27.6%] vs 19 youths [13.0%]; *P* < .001) or 3 or more (28 youths [19.3%] vs 14 youths [9.6%]; *P* = .003) dependence symptom thresholds for combustible cigarette dependence than for e-cigarette dependence (Figure 2B; eTable 4 in the Supplement). Within each tobacco product, the most common (craving) and least common (concentration problems when abstinent) symptoms were similar for combustible and e-cigarette dependence, so that cross-symptom prevalence profile shapes were similar for the 2 products. Across products, the prevalence of dependence symptoms were greater for combustible cigarettes than e-cigarettes, but the difference was only statistically significant for strong cravings to use (34 youths [24.5%] vs 19 youths [13.4%]; *P* = .002), feeling the need to use (40 youths [29.0%] vs 14 youths [9.9%], *P* < .001), and strong need or urge to use when abstinent (22 youths [16.2%] vs 12 youths [8.5%]; *P* = .01) (Figure 2B; eTable 4 in the Supplement). For descriptive purposes, comparisons of e-cigarette dependence symptoms between past-year dual users of combustible and e-cigarettes vs e-cigarette–only users are reported in Figure 2C.

#### e-Cigarette Dependence Symptoms Stratified by Key Subclassifications

The prevalence of e-cigarette dependence symptoms was higher in baseline past-year dual users of combustible and e-cigarettes compared with e-cigarette–only users (24 youths [16.4%] vs 28 youths [9.4%]; *P* = .04), users of e-cigarettes with nicotine compared with those who used e-cigarettes without nicotine (42 youths [15.2%] vs 10 youths [6.0%]; *P* = .004), and youth who used e-cigarettes in the past month compared with those who did not (41 youths [17.6%] vs 11 youths [5.2%]; *P* < .001) (Table 2). The prevalence of reporting 2 or more or 3 or more e-cigarette dependence symptoms was similarly significantly higher in these subgroups: youth with past-year combustible cigarette use, compared with youth without past-year combustible cigarette use, were more likely to report 2 or more symptoms (19 youths [13.0%] vs 15 youths [5.0%]; *P* = .004) or 3 or more symptoms (14 youths [9.6%] vs 8 youths [2.7%]; *P* = .004); youth with vaping use in the past 30 days, compared with youth without past 30 days vaping use, were more likely to report 2 or more symptoms (28 youths [12.0%] vs 6 youths [2.8%]; *P* < .001); and youth who used e-cigarettes with nicotine, compared with those who use e-cigarettes without nicotine, were more likely to report 2 or more symptoms (31 youths [11.2%] vs 3 youths [1.8%]; *P* < .001) or 3 or more symptoms (20 youths [7.2%] vs 2 youths [1.2%]; *P* = .003). Among 146 past-year cigarette and e-cigarette dual users, 145 youths had age of tobacco product use onset data. Among these 145 dual-users, 77 youths (53.1%) reported earlier e-cigarette use onset than cigarette use onset; 32 youths (22.1%) reported same age at onset for both products, and 36 youths (24.8%) reported earlier combustible cigarette use onset than e-cigarette use onset. No association was found of combustible and e-cigarette use onset sequence with e-cigarette dependence symptom status.

#### Prospective Associations Between e-Cigarette Dependence Symptoms and Subsequent Vaping

Reporting 1 or more e-cigarette dependence symptoms at baseline was associated with greater odds of any vaping during 6 months of follow-up (odds ratio, 2.56 [95% CI, 1.34–5.01]; *P* = .005) and more frequent and intense past-month nicotine vaping, including more nicotine vaping days (rate ratio [RR], 2.50 [95% CI, 1.76–3.57]; *P* < .001), sessions per vaping day (RR, 2.73 [95% CI, 1.84–4.06]; *P* < .001), and puffs per session (RR, 1.73 [95% CI, 1.14–2.62]; *P* = .01) 6 months later in models adjusted for baseline vaping characteristics (Table 3). After adjusting for the e-cigarette dependence propensity score covariate derived from a model using all covariates listed in Table 1, reporting 1 or more symptom of e-cigarette dependence remained significantly associated with greater odds of any vaping during 6 months of follow-up (adjusted odds ratio, 2.30 [95% CI, 1.07–4.94]; *P* = .02) and more nicotine vaping days (adjusted RR, 2.17 [95% CI, 1.44–3.28]; *P* < .001), sessions (adjusted RR, 2.41 [95% CI, 1.52–3.83]; *P* < .001), and puffs per session (adjusted RR, 1.70 [95% CI, 1.09–2.66]; *P* = .02) (Table 3).

#### Sensitivity and Supplemental Analyses

Sensitivity analyses of cross-tobacco product comparisons of dependence prevalence, severity, and symptom patterns that adjusted for age at onset and past 30-day use frequency of the 2 respective products yielded results that were similar to the primary results (eTable 4 in the Supplement). Additional cross-product analyses restricted to dual users who vaped nicotine found differences on the same dependence outcomes as the primary results, although differences were less robust (eTable 5 in the Supplement). Youth who use multiple tobacco products may have difficulty distinguishing the source of dependence symptoms; however, baseline past-year combustible cigarette use did not significantly moderate associations of e-cigarette dependence symptoms with subsequent vaping at 6-month follow-up (eTable 6 in the Supplement).

For descriptive purposes to examine whether results generalized across sex and nonnicotine substance use, tests of differences in cross-sectional and prospective analyses stratified by sex and number of nonnicotine substances used are reported in eTables 7, 8, 9, and 10 in the Supplement. They did not show marked differences by sex and concomitant substance use. Past-month e-cigarette and combustible cigarette use patterns by past-month nicotine vaping days are presented in eTable 11 in the Supplement for descriptive purposes.

Associations with additional behavioral health outcomes were tested for exploratory purposes, and analyses found that e-cigarette dependence was significantly associated with increases in ADHD symptom level at 6-month follow-up (β = 0.32 [95% CI, 0.07–0.57]; *P* = .01); this association was amplified by the number of other substances used (β per additional substance = 0.22 [95% CI, 0.03–0.41]; *P* = .02) (eTable 12 in the Supplement). Additionally, reporting 1 or more e-cigarette dependence symptoms at baseline was associated with heavier combustible cigarette smoking at follow-up, including more cigarette smoking days (adjusted RR, 2.33 [95% CI, 1.56–3.52]; *P* < .001) and more cigarettes smoked per day (adjusted RR, 3.03 [95% CI, 1.82–5.04]; *P* < .001) (eTable 13 in the Supplement).

## Discussion

This cohort study provides some of the most detailed evidence to date on the prevalence and symptom presentation of e-cigarette dependence and its association with future e-cigarette use among youth, to our knowledge. Previous studies have provided less comprehensive characterizations of e-cigarette dependence, without comparisons with combustible cigarette dependence.^6–8^ To our knowledge, this is also the first prospective longitudinal investigation of the association of e-cigarette dependence symptoms with subsequent vaping patterns. Our results suggest that e-cigarette dependence symptoms may be associated with future vaping patterns.

In this study, the prevalence of e-cigarette dependence symptoms was relatively low (reported by 11.7% of past-year e-cigarette users and 17.6% of past-month users) and, consistent with prior research,^7^ primarily characterized by cravings and a perceived need to vape. Although prevalence and severity of dependence symptoms were approximately 2-fold as large for combustible cigarettes as e-cigarettes in users of both products, the most common (ie, cravings, urges) and least common (ie, difficulty concentration when abstaining from e-cigarettes) symptoms were similar across the 2 products, as were the qualitative profiles across all 10 symptoms. Importantly, the cross-product comparisons were conducted within-persons among dual users, eliminating confounding differences between smokers and vapers. Unlike combustible cigarettes, e-cigarettes and e-liquids vary in nicotine content and delivery.^3^ Few youth in this study likely used the now-popular pod mod–style e-cigarette products (eg, JUUL) that deliver large amounts of nicotine efficiently.^21^ Considering the elevated dependence symptoms reported among youth who vaped nicotine in this study despite low probability of pod mod use, these results suggest that e-cigarette dependence may be of notable clinical and public health significance.

Electronic cigarette dependence symptoms were elevated in certain subgroups expected to be at higher risk, including youth who vaped recently, used e-cigarettes that contained nicotine, or used both e-cigarettes and combustible cigarettes. A 2018 study^22^ found that dual users had higher levels of nicotine biomarkers than users of e-cigarettes only. To our knowledge, this is among the first investigations to find that e-cigarette dependence symptoms were experienced even among youth who reported using only e-cigarettes without nicotine—a sizeable proportion of youth e-cigarette users.^5^ Nicotine-containing products may have been mislabeled, or youth with histories of nicotine vaping in early adolescence who later switched to only nicotine-free vaping over the past year may have experienced cravings triggered by cues associated with the act of vaping.

Youth who used e-cigarettes and reported at least 1 e-cigarette dependence symptom were more likely to continue vaping and to vape more frequently and intensely 6 months later than their peers who did not report any dependence symptoms. This finding is consistent with a 2019 study^23^ that suggested that vaping continuation, with escalation of use frequency and dependence symptoms, is common. Our study further suggests that youth with dependence symptoms are at elevated risk for continuation and escalation. Symptoms of e-cigarette dependence (eg, craving) may directly increase motivation to use, and increased use may recapitulate a cycle of worsening dependence. Although our observational study design precludes such causal inferences, findings suggest that e-cigarette dependence may be associated with subsequent vaping patterns even after adjusting for dependence propensity defined by numerous potentially confounding influences.

### Implications

In clinical settings, e-cigarette dependence symptom screening questions may identify youth at risk for vaping progression who may benefit from intervention. In regulatory decision-making, dependence is a potential health consequence of e-cigarette use that should be considered. Adolescents are particularly vulnerable to nicotine exposure,^1,2^ and our findings suggest that dependence symptoms associated with nicotine exposure via e-cigarettes are associated with greater risk for escalation of vaping behavior. The development of dependence in youth is an important public health consequence that should not be overlooked.

### Limitations

This study has some limitations. First, all participants were recruited from high schools in Los Angeles, California; therefore, extension to different regions would be informative. Second, data were collected in the 2016 to 2017 school year, before high-nicotine e-cigarettes (eg, pod mods) became popular among youth.^24^ Third, all measures were self-reported and did not include clinical diagnosis of nicotine dependence. While the measure of dependence was selected for its presumed applicability to both tobacco products and its ability to capture key features of the dependence syndrome (eg, cravings or urges to use, loss of autonomy over use),^25^ other measures of e-cigarette dependence that are correlated with nicotine exposure merit inclusion in future research to address varying aspects of the dependence syndrome.^6^ Fourth, the follow-up period was limited to 6 months, leaving unclear the long-term association of e-cigarette dependence with future use.

## Conclusions

In this cohort of youth who used e-cigarettes in the past year, e-cigarette dependence symptoms were similar in nature to combustible cigarette dependence but less prevalent and severe. Symptoms were elevated in subgroups presumed to be at increased risk, including youth who recently vaped, vaped nicotine, and also smoked, and were associated with increased risk of future vaping. These findings suggest that youth who report vaping may benefit from screening for e-cigarette dependence symptoms, such as cravings and perceived need to vape—symptoms that may be associated with risk of future vaping persistence and progression. Dependence is an important health effect of e-cigarette use that should be considered in federal regulatory decisions that weigh the relative harms and benefits of e-cigarettes.

## Supplementary Material

SM

## Figures and Tables

**Table 1.** Participant Characteristics in Overall Sample and Stratified by e-Cigarette Dependence Symptom Status at Baseline

	Youths, No. (%) [Total Respondents, No.]			
		e-Cigarette Dependence Symptoms Reported		
Variable	Overall Sample (N = 444)	0 (n = 392)	≥1 (n = 52)	*P* Value for Contrast^a^

Female	217 (48.9) [444]	194 (49.5) [392]	23 (44.2) [52]	.55

Age, mean (SD), y	17.48 (0.39) [444]	17.47 (0.37) [392]	17.54 (0.49) [52]	.23

Race/ethnicity				

Hispanic	187 (42.9) [436]	168 (43.8) [384]	19 (36.5) [52]	.61

Asian	76 (17.4) [436]	68 (17.7) [384]	8 (15.4) [52]	

African American	14 (3.2) [436]	11 (2.9) [384]	3 (5.8) [52]	

White	87 (20.0) [436]	74 (19.3) [384]	13 (25.0) [52]

Other^b^	72 (16.5) [436]	63 (16.4) [384]	9 (17.3) [52]

Parents graduated college^c^	207 (52.3) [396]	181 (51.6) [384]	26 (57.8) [45]	.53

Age at e-cigarette use onset, mean (SD), y	15.31 (1.58) [440]	15.33 (1.55) [388]	15.15 (1.81) [52]	.43

Any past 6-mo e-cigarette use	265 (60.1) [441]	224 (57.4) [390]	41 (80.4) [51]	.001

Past 30-d, mean (SD)				

Nicotine vaping days, No.	1.74 (5.71) [441]	1.17 (4.35) [390]	6.10 (10.85) [51]	<.001

Nicotine vaping sessions/d, No.	1.55 (4.43) [438]	1.05 (3.60) [386]	5.25 (7.42) [52]	<.001

Puffs per nicotine vaping sessions, No.	1.42 (3.83) [440]	0.96 (2.89) [388]	4.87 (7.04) [52]	<.001

Age at combustible cigarette use onset, mean (SD), y	15.04 (2.34) [226]	15.14 (2.22) [194]	14.45 (2.95) [32]	.12

Any past-year combustible cigarette smoking	146 (32.9) [444]	122 (31.1) [392]	24 (46.2) [52]	.04

Past 30-d, mean (SD)				

Cigarette smoking days, No.	1.19 (4.64) [438]	1.06 (4.57) [387]	2.16 (5.07) [51]	.11

Cigarettes smoked/d, No.	0.49 (2.19) [436]	0.39 (1.85) [385]	1.29 (3.81) [51]	.005

Ever use cigar, hookah, or smokeless tobacco	287 (65.2) [440]	244 (62.7) [389]	43 (84.3) [51]	.002

Ever substance use				

Alcohol	402 (91.8) [438]	353 (91.2) [387]	49 (96.1) [51]	.41

Smoked cannabis	340 (77.8) [437]	296 (76.7) [386]	44 (86.3) [51]	.15

Vaped cannabis	212 (48.3) [439]	180 (46.4) [389]	32 (62.7) [51]	.04

Other drugs^d^	241 (54.8) [440]	207 (53.2) [388]	34 (66.7) [51]	.07

Mental health symptoms, mean (SD), No.				

Conduct problems^e^	15.93 (6.88) [426]	15.60 (6.34) [376]	18.34 (9.85) [50]	.008

ADHD^f^	12.53 (10.20) [427]	12.26 (9.83) [376]	14.50 (12.49) [51]	.14

Social phobia^g^	9.25 (7.29) [433]	9.10 (7.17) [382]	10.33 (8.10) [51]	.26

Major depressive disorder^g^	8.16 (7.23) [433]	7.65 (6.97) [382]	11.92 (8.03) [51]	<.001

Generalized anxiety disorder^g^	6.91 (4.70) [435]	6.74 (4.70) [384]	8.20 (4.60) [51]	.04

Mania^h^	5.44 (3.68) [438]	5.23 (3.64) [387]	7.04 (3.61) [51]	.001

Panic disorder^g^	4.24 (5.31) [436]	4.05 (5.14) [384]	5.63 (6.28) [52]	.04

Obsessive-compulsive disorder^g^	3.19 (3.33) [439]	3.08 (3.29) [387]	4.02 (3.57) [52]	.06

Abbreviations: ADHD, attention-deficit/hyperactivity disorder; e-cigarette, electronic cigarette.

a Calculated using the χ^2^ test for categorical variables and 1-way analysis of variance for continuous variables.

b Includes American Indian or Alaskan Native, Native Hawaiian or Pacific Islander, multiethnic or multiracial, or other.

c Excludes 48 youths who did not respond to the survey question or who marked don’t know.

d Includes inhalants, cocaine, methamphetamine, lysergic acid diethylamide (LSD), ecstasy, heroin, salvia, prescription pain medications, tranquilizers or sedatives, diet pills, prescription stimulants, and bath salts.

e Calculated by summing scores (range, 11–66) for 11 delinquent behaviors indicative of conduct problems over the past 6 months; each behavior is rated from 1 (never) to 6 (10 or more times), with higher scores indicating greater frequency of engaging in these behaviors.

f Symptom sum score of ADHD symptoms self-rating scale (range, 0–54); higher scores indicate more frequent ADHD symptoms in the past 6 months.

g Symptom sum scores from the Revised Child Anxiety and Depression Scales major depressive disorder (range, 0–30), generalized anxiety disorder (range, 0–18), panic disorder (range, 0–27), social phobia (range, 0–27), and obsessive-compulsive disorder (range, 0–18) subscales; higher scores indicate more severe mental health symptoms.

h Symptom sum from the 15-item Mood Disorder Questionnaire (range, 0–13); higher scores indicate more past-year mania symptoms.

**Table 2.** Electronic Cigarette Dependence Symptoms Stratified by Nicotine, Past 30-Day Vaping, and Past-Year Combustible Cigarette Use

Vaping Dependence Symptoms Reported	Use of Electronic Cigarettes With Nicotine in Past y			Vaped in Past 30 d			Combustible Cigarette Use in Past y		
	Youths, No. (%)		Test of Difference, *P* Value^a^	Youths, No. (%)		Test of Difference, *P* Value^a^	Youths, No. (%)		Test of Difference, *P* Value^a^
	No (n = 168)	Yes (n = 276)		No (n = 211)	Yes (n = 233)		No (n = 298)	Yes (n = 146)	

≥1	10 (6.0)	42 (15.2)	.004	11 (5.2)	41 (17.6)	<.001	28 (9.4)	24 (16.4)	.04

≥2	3 (1.8)	31 (11.2)	<.001	6 (2.8)	28 (12.0)	<.001	15 (5.0)	19 (13.0)	.004

≥3	2 (1.2)	20 (7.2)	.003	6 (2.8)	16 (6.9)	.08	8 (2.7)	14 (9.6)	.004

a Calculated by χ^2^ tests.

**Table 3.** Association of Baseline Electronic Cigarette Dependence Symptoms With Subsequent Vaping Patterns at 6-Month Follow-up^a^

	Mean (SD)		Unadjusted		Adjusted^b^	
Outcome	0 Dependence Symptoms at Baseline (n = 392)	≥1 Dependence Symptom at Baseline (n = 52)	Association Estimate (95% CI)	*P* Value	Association Estimate (95% CI)	*P* Value

Vaped during follow-up, No. (%)	152 (38.8)	32 (61.5)	2.56 (1.34–5.01)^c^	.005	2.30 (1.07–4.94)^c^	.02

Past 30-d						

No. of nicotine vaping days^d^	1.92 (5.61)	7.88 (11.74)	2.50 (1.76–3.57)^e^	<.001	2.17 (1.44–3.28)^e^	<.001

No. of nicotine vaping sessions/vaping day^f^	1.21 (3.74)	5.22 (8.06)	2.73 (1.84–4.06)^e^	<.001	2.41 (1.52–3.83)^e^	<.001

Puffs/nicotine vaping session^f^	0.97 (2.62)	3.69 (5.97)	1.73 (1.14–2.62)^e^	.01	1.70 (1.09–2.66)^e^	.02

a Regression models of association between electronic cigarette dependence status and respective outcome at follow-up with baseline status on the respective outcome as a covariate using a complex design accounting for clustering of data within schools.

b Adjusted for a propensity score for vaping dependence status derived from youth sex, age, race/ethnicity, parental education level, electronic cigarette use onset age, past year combustible cigarette smoking, past 30-day number of days smoked cigarettes, past 30-day number of cigarette smoked per day, ever use of cigar, hookah, or smokeless tobacco, ever alcohol use, ever smoked cannabis, ever vaped cannabis, ever other drug use, major depressive disorder, generalized anxiety disorder, panic disorder, social phobia, obsessive compulsive disorder, mania, attention-deficit/hyperactivity disorder, and conduct problems.

c Odds ratio (95% CI) from logistic regression model for binary outcomes.

d Response range, 0 to 30.

e Rate ratio (95% CI) from negative binomial model for count outcomes.

f Response range, 0 to 20.

## Key Points

### Question

What are the prevalence, symptom presentation, and future vaping patterns associated with youth electronic cigarette (e-cigarette) dependence?

### Findings

In this cohort study of 444 youths aged 16 to 18 years with past-year e-cigarette use, 11.7% of youth reported experiencing at least 1 e-cigarette dependence symptom. While less common and severe than combustible cigarette dependence, e-cigarette dependence had a similar symptom profile and was positively associated with vaping continuation, frequency, and intensity 6 months later.

### Meaning

These findings suggest that e-cigarette dependence among youth may be an expression of tobacco use disorder that is associated with use escalation.
