# Supplementary material for: Prevalence of Electronic Cigarette Dependence Among Youth and Its Association With Future Use
Source: JAMA Netw Open. Author manuscript; Available in PMC 2021 Feb 5. (PMC7780897; doi:10.1001/jamanetworkopen.2019.21513)
Supplement: SM [file NIHMS1653187-supplement-SM.pdf]

Supplementary Online Content

Vogel EA, Cho J, McConnell RS, Barrington-Trimis JL, Leventhal AM. Prevalence of electronic cigarette dependence among youth and its association with future use. *JAMA Netw Open*. 2020;3(2):e1921513. doi:10.1001/jamanetworkopen.2019.21513

eAppendix. Supplementary Methods
eTable 1. Sociodemographic Characteristics of Included and Excluded Youth in the Primary Analytic Sample
eTable 2. Descriptive Data on Electronic Cigarette Use Patterns Stratified by Use of Electronic Cigarettes With vs Without Nicotine in Past Year
eTable 3. Frequency Distribution of Total Number of Tobacco Product Dependence Symptoms
eTable 4. Electronic Cigarette Dependence Symptoms in Overall Sample and Comparison of Electronic Cigarette and Combustible Cigarette Dependence Symptoms in Past Year Dual Users of Both Products
eTable 5. Electronic Cigarette Dependence Symptoms in Overall Sample and Comparison of Electronic Cigarette and Combustible Cigarette Dependence Symptoms in Past Year Dual Users
eTable 6. Interaction of Baseline Past-Year Combustible Cigarette Use as a Moderator in Associations Between Electronic Cigarette Dependence Symptoms and Subsequent Vaping at 6-Month Follow-up
eTable 7. Vaping Dependence Symptoms Stratified by Nicotine, Past 30-Day Vaping, and Past Year Combustible Cigarette Use Status Stratified by Sex
eTable 8. Vaping Dependence Symptoms Stratified by Nicotine, Past 30-Day Vaping, and Past Year Combustible Cigarette Use Status Stratified by Number of Substances Used
eTable 9. Interaction of Sex as a Moderator in Associations Between Electronic Cigarette Dependence Symptoms and Subsequent Vaping at 6-Month Follow-up
eTable 10. Interaction of Baseline Number of Nonnicotine Substances Used as a Moderator in Associations Between Electronic Cigarette Dependence Symptoms and Subsequent Vaping At 6-Month Follow-up
eTable 11. Past 30-Day Electronic Cigarette and Combustible Cigarette Use Patterns by Past 30-Day Number of Nicotine Vaping Days at Each Assessment
eTable 12. Interaction Effects of Electronic Cigarette Dependence Status and Other Drug Use at Baseline on Attention-Deficit/Hyperactivity Disorder Symptom Level at Follow-up
eTable 13. Prospective Associations of Baseline Electronic Cigarette Dependence Status With Combustible Cigarette Use Outcomes at Follow-up
eReferences.

This supplementary material has been provided by the authors to give readers additional information about their work.

© 2020 Vogel EA et al. *JAMA Network Open.*

eAppendix. Supplementary Methods

Supplemental Information of Vaping and Mental Health Variables Assessment

Outcomes for longitudinal analysis. At baseline and follow-up assessments, four e-cigarette use variables were assessed. First, two separate questions were used to assess past 6-month use of: (1) "e-cigarettes for nicotine" and (2) "e-cigarettes without nicotine or hash oil".[1] Participants who responded affirmatively to either e-cigarette question (or both) were classified as past 6-month e-cigarette users. Participants reported their past 30-day frequency and daily intensity of e-cigarette use. The number of days they used e-cigarette with nicotine in the past 30 days was measured (response options: 0, 1-2, 3-5, 6-9, 10-14, 15-19, 20-24, 25-29, all 30 days). These response categories were transformed into quantitative count variables by taking the mean integer value of each ordinal past 30-day frequency variable as in past work[2]: 0, 2, 4, 8, 12, 17, 22, 27, 30. For the daily intensity variables, participants reported the number of nicotine vaping sessions on each vaping day (response options: 0, 1, 2, 3-5, 6-9, 10-14, 15-20, >20 times per day) as well as the number of puffs per nicotine vaping sessions (response options: 0, 1, 2, 3-5, 6-9, 10-14, 15-20, >20 puffs). These response categories were also transformed into quantitative count variables by taking the lowest value of each ordinal category (i.e., 0, 1, 2, 3, 6, 10, 15, 20) for use in the daily intensity analyses, consistent with prior work.[3]

Mental health. Students were administered the Revised Child Anxiety and Depression Scales (RCADS),[4] which instructs respondents to rate the frequency with which they experienced Diagnostic and Statistical Manual of Mental Disorders–4th Edition[5] emotional disorder symptoms on a 4-point scales (0 [Never] to 3 [Always]). The RCADS yields separate subscale sum severity scores for major depressive disorder (MDD; 10 items; Cronbach's α in this sample = .94), generalized anxiety disorder (GAD; 6 items; α = .91), social phobia (SP; 9 items; α = .93), panic disorder (PD; 9 items; α = .93), and obsessive compulsive disorder (OCD; 6 items; α = .86). Past year mania was assessed using the Mood Disorder Questionnaire (MDQ).[6] The MDQ has 15 items (0=no, 1=yes), which reflect different DSM-IV manic or hypomanic symptoms or behaviors, which were summed (α = .78). Attention-Deficit/Hyperactivity Disorder (ADHD) symptom was measured using the Current Symptoms Self-Report Form[7] measure of the DSM-IV criteria, which include nine inattention symptoms (e.g., difficulty organizing and completing tasks) and nine hyperactivity-impulsivity symptoms (e.g., trouble remaining still or with task persistence). Respondents rated the frequency that they experienced each symptom in the preceding 6 months: never or rare (=0), sometimes (=1), often (=2), or very often (=3); responses to the 18 items are summed (α = .93). Conduct problems were measured with a sum of frequency ratings for engaging in 11 behaviors (e.g., 'stealing,' 'lying to parents'; each item rated: 1 [never] to 6 [≥10 times]; α = .79) in the past 6-months.[8]

E-cigarette Dependence Risk Propensity Score Calculation

Adjustment for a risk propensity score is a desirable alternative to conventional multi-variable simultaneous covariate adjustment, which can result in unstable model estimates and model "overfitting" when a large number of covariates area are considered.[9-11] The propensity score variable was derived from a prediction model in which baseline e-cigarette dependence status regressed on all 25 baseline variables presented in Table 1 (i.e., youth gender, age, race/ethnicity, parental education level, e-cigarette use onset age, past year combustible cigarette smoking, past 30-day number of days smoked cigarettes, past 30-day number of cigarette smoked per day, ever use of cigar, hookah, or smokeless tobacco, ever alcohol use, ever smoked cannabis, ever vaped cannabis, ever other drug use, major depressive disorder, generalized anxiety disorder, panic disorder, social phobia, obsessive compulsive disorder, mania, ADHD, and conduct problems). Onset age of combustible cigarette use is reported in Table 1, but was not included in the propensity score because only dual users provided this information. A propensity score was then calculated from the estimates derived from the logistic regression model including the 25 covariates. Adjusted models presented in Table 3 includes the propensity score as a covariate.

© 2020 Vogel EA et al. *JAMA Network Open.*

eTable 1. Sociodemographic Characteristics of Included and Excluded Youth in the Primary Analytic Sample

	Excluded from analytic sample (N=2,952)						
	**Not surveyed at fall 12th grade (N=228)[a]**	**No past year e-cigarette use (N=2520)[b]**	**Did not answer e-cigarette use questions (N=7)[c]**	**Administered abbreviated survey that omitted vaping dependence items (N=181)[d]**	**Not surveyed at 6-month follow-up (N=16)[e]**	**Primary analytic sample (N=444)[f]**	**Test of overall group difference[g]**
**Variable**	**N (%) / *M* (*SD*)**	**N (%) / *M* (*SD*)**	**N (%) / *M* (*SD*)**	**N (%) / *M* (*SD*)**	**N (%) / *M* (*SD*)**	**N (%) / *M* (*SD*)**	**P-value**
Female (vs. Male), N (%)	86 (37.9)[3]	1417 (56.2)[1]	2 (28.6)[3,4]	85 (47.2)[2,3]	5 (31.3)[3,4]	218 (49.1)[2]	<.001
Age, year, *M* (*SD*)	17.70 (0.45)[1,2]	17.49 (0.41)[3]	17.80 (0.55)[1,2]	17.59 (0.43)[2]	17.65 (0.54)[1,2]	17.48 (0.39)[2,3]	<.001
Race/ethnicity, N (%)							
Hispanic	107 (49.3)[2]	1212 (48.9)[2]	5 (83.3)[1]	87 (50.6)[2]	7 (43.8)[2]	187 (42.9)[2]	
Asian	18 (8.3)[2]	448 (18.1)[1]	0 (0.0)[2]	17 (9.9)[2]	1 (6.3)[2]	76 (17.4)[1]	<.001
African American	19 (8.8)[1]	125 (5.0)[1,2]	0 (0.0)[2]	6 (3.5)[2]	1 (6.3)[1,2]	15 (3.4)[2]	
White	37 (17.1)[1,2]	378 (15.2)[2]	1 (16.7)[1,2]	34 (19.8)[1,2]	6 (37.5)[1]	88 (20.2)[1,2]	
Other[h]	36 (16.6)[1]	316 (12.7)[1]	0 (0.0)[1]	28 (16.3)[1]	1 (6.3)[1]	70 (16.1)[1]	
Parents graduated college, N (%)[i]	85 (45.9)[1]	1112 (51.1)[1]	3 (60.0)[1]	67 (44.1)[1]	7 (43.8)[1]	208 (52.4)[1]	.37

[a]Available data (Ns = 185 – 227).
[b]Available data (Ns = 2176 – 2520).
[c]Available data (Ns = 5 – 7).
[d]Available data (Ns = 152 – 181).
[e]Available data (Ns = 15 – 16).
[f]Available data (Ns = 397 – 444).
[g]Calculated using the χ^2 test for categorical variables and the one-way Analysis of Variance (ANOVA) for continuous variables.
[h]Other race/ethnicity = students who selected 'American Indian/Alaskan Native,' 'Native Hawaiian/Pacific Islander,' 'Multiethnic/Multiracial,' or 'Other' options for the forced-choice race/ethnicity question.
[i]Students (N=465) who did not respond to the survey question or who marked "don't know" are not included in the denominator.
*Statistically significant after Benjamini-Hochberg corrections for multiple testing to control false discovery rate at .05 (based on two-tailed corrected P-value).

eTable 2. Descriptive Data on Electronic Cigarette Use Patterns Stratified by Use of Electronic Cigarettes With vs Without Nicotine in Past Year

**Vaping at 6-month follow-up**		**Use of e-cigarettes with nicotine in past year (N=444)[a]**		
		**No (N=168)[a]**	**Yes (N=276)[a]**	**Test of Difference[b]**
Any past 6-month e-cigarette use, N (%)		44 (26.2)	140 (50.7)	<.001
Past 30-day number of nicotine vaping days, *M* (*SD*)		0.38 (1.92)	4.01 (8.34)	<.001
Past 30-day number of nicotine vaping sessions, *M* (*SD*)		0.20 (0.91)	2.59 (5.66)	<.001
Past 30-day puffs per nicotine vaping sessions, *M* (*SD*)		0.38 (1.30)	1.84 (3.97)	<.001

[a]Available data Ns for denominator: Overall sample (Ns = 439 – 444), No (N = 168), and Yes (Ns = 271 – 276).
[b]Calculated using the χ^2 test for categorical variables and one-way Analysis of Variance (ANOVA) for continuous variables.

© 2020 Vogel EA et al. *JAMA Network Open.*

eTable 3. Frequency Distribution of Total Number of Tobacco Product Dependence Symptoms

	Any past year e-cigarette users (N=444)[a]		Past year dual users of e-cigarettes and combustible cigarettes (N=146)[a]	
**Dependence Symptoms for Respective Product**	**E-Cigarette Dependence**		**E-Cigarette Dependence**	**Combustible Cigarette Dependence**
	**N (%)**		**N (%)**	**N (%)**
0 symptoms	392 (88.3)		122 (83.6)	102 (72.3)
1 symptom	18 (4.1)		5 (3.4)	3 (2.1)
2 symptoms	12 (2.7)		5 (3.4)	12 (8.3)
3 symptoms	7 (1.6)		1 (0.7)	8 (5.5)
4 symptoms	7 (1.6)		6 (4.1)	2 (1.4)
5 symptoms	1 (0.2)		0 (0.0)	6 (4.1)
6 symptoms	2 (0.5)		1 (0.7)	3 (2.1)
7 symptoms	2 (0.5)		2 (1.4)	3 (2.1)
8 symptoms	0 (0.0)		0 (0.0)	0 (0.0)
9 symptoms	0 (0.0)		0 (0.0)	3 (2.1)
10 symptoms	5 (1.1)		4 (2.7)	3 (2.1)

[a]Available data Ns for the respective variable and denominator for percentage.

© 2020 Vogel EA et al. *JAMA Network Open.*

eTable 4. Electronic Cigarette Dependence Symptoms in Overall Sample and Comparison of Electronic Cigarette and Combustible Cigarette Dependence Symptoms in Past Year Dual Users of Both Products

Dependence Symptoms for Respective Product	Any past year e-cigarette users (N=444)	Past year dual users of e-cigarettes and combustible cigarettes (N=146)			
	E-Cigarette Dependence	E-Cigarette Dependence	Combustible Cigarette Dependence	Within-Person Difference, P-Value	
	N (%)[a]	N (%)[b]	N (%)[b]	Unadjusted[c]	Adjusted[d]
1 or more symptoms	52 (11.7)	24 (16.4)	43 (29.7)	.002	<.001
2 or more symptoms	34 (7.7)	19 (13.0)	40 (27.6)	<.001	<.001
3 or more symptoms	22 (5.0)	14 (9.6)	28 (19.3)	.004	.003
Individual symptoms					
Tried to quit, but couldn't	13 (3.0)	8 (5.6)	12 (8.5)	.42	.15
Use now because it difficult to quit	11 (2.5)	8 (5.6)	12 (8.5)	.39	.17
Felt addicted to product	18 (4.2)	11 (7.9)	20 (14.1)	.14	.06
Strong cravings to use	38 (8.9)	19 (13.4)	34 (24.5)	.009	.002
Felt like needed to use	25 (5.8)	14 (9.9)	40 (29.0)	<.001	<.001
Hard to keep from use where not permitted	14 (3.3)	9 (6.2)	12 (8.6)	.73	.10
Withdrawal symptoms after abstaining					
Difficulty concentrating	7 (1.6)	6 (4.3)	7 (5.0)	.98	.59
Irritability	11 (2.6)	8 (5.7)	14 (10.1)	.06	.05
Strong need or urge to use	16 (3.7)	12 (8.5)	22 (16.2)	.02	.01
Nervous, restless, or anxious	8 (1.9)	7 (5.1)	12 (8.7)	.11	.11

[a]Available data Ns for the respective variable and denominator for percentage = 420 – 444.
[b]Available data Ns for the respective variable and denominator for percentage = 135 – 146.
[c]Within-person difference between e-cigarette and combustible cigarette dependence for respective outcome based McNemar's tests.
[d]Within-person difference between e-cigarette and combustible cigarette dependence for respective outcome after adjusting for baseline age of use onset and number of days used in the past 30 for respective product using a general estimating equation model.

© 2020 Vogel EA et al. *JAMA Network Open.*

eTable 5. Electronic Cigarette Dependence Symptoms in Overall Sample and Comparison of Electronic Cigarette and Combustible Cigarette Dependence Symptoms in Past Year Dual Users

**Dependence Symptoms for Respective Product**	**Past year dual users of e-cigarettes with nicotine and combustible cigarettes (N=120)**				
	**E-Cigarette Dependence**	**Combustible Cigarette Dependence**		**Within-Person Difference, P-Value**	
	**N (%)[a]**	**N (%)[b]**		**Unadjusted[c]**	**Adjusted[d]**
1 or more symptoms	24 (20.0)	39 (32.8)		.009	.001
2 or more symptoms	19 (15.8)	36 (30.3)		.002	<.001
3 or more symptoms	14 (11.7)	24 (20.2)		.03	.02
Individual symptoms					
Tried to quit, but couldn't	8 (6.8)	11 (10.3)		.58	.22
Use now because it difficult to quit	8.(6.8)	12 (10.3)		.39	.18
Felt addicted to product	11 (9.6)	18 (15.5)		.30	.07
Strong cravings to use	19 (16.4)	30 (26.3)		.04	.01
Felt like needed to use	14 (12.1)	36 (31.9)		<.001	<.001
Hard to keep from use where not permitted	9 (8.0)	11 (9.6)		.73	.15
Withdrawal symptoms after abstaining					
Difficulty concentrating	6 (5.2)	7 (6.1)		.99	.58
Irritability	8 (7.0)	12 (10.6)		.18	.12
Strong need or urge to use	12 (10.4)	19 (17.0)		.06	.04
Nervous, restless, or anxious	7 (6.3)	11 (9.7)		.18	.16

[a]Available data Ns for the respective variable and denominator for percentage = 112 – 120.
[b]Available data Ns for the respective variable and denominator for percentage = 112 – 119.
[c]Within-person difference between e-cigarette and combustible cigarette dependence for respective outcome based McNemar's tests.
[d]Within-person difference between e-cigarette and combustible cigarette dependence for respective outcome after adjusting for baseline age of use onset and number of days used in the past 30 for respective product using a general estimating equation model.

© 2020 Vogel EA et al. *JAMA Network Open.*

eTable 6. Interaction of Baseline Past-Year Combustible Cigarette Use as a Moderator in Associations Between Electronic Cigarette Dependence Symptoms and Subsequent Vaping at 6-Month Follow-up

E-cigarette use outcome at follow-up	Association of baseline e-cigarette dependence status × past-year combustible cigarette use interaction term with outcome at follow-up[a]		
	Unadjusted[b]		Adjusted[c]
	P-Value		P-Value
Any vaping over follow-up	.31		.54
Past 30-day number of nicotine vaping days	.12		.24
Past 30-day number of nicotine vaping sessions	.26		.49
Past 30-day puffs per nicotine vaping sessions	.08		.10

[a]Regression models of association between the interaction term of the hooked on nicotine checklist for e-cigarette dependence (≥1 vs. 0 symptoms) and past-year combustible cigarette use and respective outcome with a complex design accounting for clustering of data within schools.
[b]Unadjusted models include baseline status on the respective outcome as a covariate.
[c]Adjusted models include respective baseline outcome covariates and a propensity score for vaping dependence status derived from potentially confounding variables (i.e., youth gender, age, race/ethnicity, parental education level, e-cigarette use onset age, past year combustible cigarette smoking, past 30-day number of days smoked cigarettes, past 30-day number of cigarette smoked per day, ever use of cigar, hookah, or smokeless tobacco, ever alcohol use, ever smoked cannabis, ever vaped cannabis, ever other drug use, major depressive disorder, generalized anxiety disorder, panic disorder, social phobia, obsessive compulsive disorder, mania, ADHD, and conduct problems).

© 2020 Vogel EA et al. *JAMA Network Open.*

eTable 7. Vaping Dependence Symptoms Stratified by Nicotine, Past 30-Day Vaping, and Past Year Combustible Cigarette Use Status Stratified by Sex

**FEMALE (N=217) Vaping dependence symptoms**	**Use of e-cigarettes with nicotine in past year**				**Vaped in past 30 days**				**Past year combustible cigarette use**		
	**No (N=88)[a]**	**Yes (N=129)[a]**	**Test of Difference[b]**		**No (N=114)[a]**	**Yes (N=103)[a]**	**Test of Difference[b]**		**No (N=153)[a]**	**Yes (N=64)[a]**	**Test of Difference[b]**
	**N (%)**	**N (%)**	**P-Value**		**N (%)**	**N (%)**	**P-Value**		**N (%)**	**N (%)**	**P-Value**
1 or more symptoms	5 (5.7)	18 (14.0)	.05		8 (7.0)	15 (14.6)	.07		13 (8.5)	10 (15.6)	.12
2 or more symptoms	1 (1.1)	13 (10.1)	.008*		4 (3.5)	10 (9.7)	.06		6 (3.9)	8 (12.5)	.02*
3 or more symptoms	1 (1.1)	10 (7.8)	.03*		4 (3.5)	7 (6.8)	.27		4 (2.6)	7 (10.9)	.01*
**MALE (N=227) Vaping dependence symptoms**	**Use of e-cigarettes with nicotine in past year**				**Vaped in past 30 days**				**Past year combustible cigarette use**		
	**No (N=80)[a]**	**Yes (N=147)[a]**	**Test of Difference[b]**		**No (N=97)[a]**	**Yes (N=130)[a]**	**Test of Difference[b]**		**No (N=145)[a]**	**Yes (N=82)[a]**	**Test of Difference[b]**
	**N (%)**	**N (%)**	**P-Value**		**N (%)**	**N (%)**	**P-Value**		**N (%)**	**N (%)**	**P-Value**
1 or more symptoms	5 (6.3)	24 (16.3)	.03*		3 (3.1)	26 (20.0)	<.001*		15 (10.3)	14 (17.1)	.15
2 or more symptoms	2 (2.5)	18 (12.2)	.01*		2 (2.1)	18 (13.8)	.002*		9 (6.2)	11 (13.4)	.07
3 or more symptoms	1 (1.3)	10 (6.8)	.06		2 (2.1)	9 (6.9)	.09		4 (2.8)	7 (8.5)	.05

[a]Available data Ns for the respective variable and denominator for percentage. Total analytic sample N = 444 (Past-year e-cigarette users with vaping dependence data at baseline and past 6-month e-cigarette use data at 6-month follow-up).
[b]Test of group differences from χ^2 tests.
*Statistically significant after Benjamini-Hochberg corrections for multiple testing to control false discovery rate at .05 (based on two-tailed corrected P-value).

© 2020 Vogel EA et al. *JAMA Network Open.*

eTable 8. Vaping Dependence Symptoms Stratified by Nicotine, Past 30-Day Vaping, and Past Year Combustible Cigarette Use Status Stratified by Number of Substances Used

Substance 0 (N=20) Vaping dependence symptoms	Use of e-cigarettes with nicotine in past year			Vaped in past 30 days			Past year combustible cigarette use		
	No (N=14)[a]	Yes (N=6)[a]	Test of Difference[b]	No (N=15)[a]	Yes (N=5)[a]	Test of Difference[b]	No (N=19)[a]	Yes (N=1)[a]	Test of Difference[b]
	N (%)	N (%)	P-Value	N (%)	N (%)	P-Value	N (%)	N (%)	P-Value
1 or more symptoms	1 (7.1)	1 (16.7)	.52	1 (6.7)	1 (20.0)	.39	2 (10.5)	0 (0.0)	.73
2 or more symptoms	0 (0.0)	1 (16.7)	.12	0 (0.0)	1 (20.0)	.08	1 (5.3)	0 (0.0)	.81
3 or more symptoms	0 (0.0)	1 (16.7)	.12	0 (0.0)	1 (20.0)	.08	1 (5.3)	0 (0.0)	.81
**Substance 1 (N=57) Vaping dependence symptoms**	**Use of e-cigarettes with nicotine in past year**			**Vaped in past 30 days**			**Past year combustible cigarette use**		
	No (N=28)[a]	Yes (N=29)[a]	Test of Difference[b]	No (N=32)[a]	Yes (N=25)[a]	Test of Difference[b]	No (N=50)[a]	Yes (N=7)[a]	Test of Difference[b]
	N (%)	N (%)	P-Value	N (%)	N (%)	P-Value	N (%)	N (%)	P-Value
1 or more symptoms	1 (3.6)	2 (6.9)	.57	2 (6.3)	1 (4.0)	.71	3 (6.0)	0 (0.0)	.51
2 or more symptoms	1 (3.6)	1 (3.4)	.98	1 (3.1)	1 (4.0)	.86	2 (4.0)	0 (0.0)	.59
3 or more symptoms	0 (0.0)	1 (3.4)	.32	1 (3.1)	0 (0.0)	.37	1 (2.0)	0 (0.0)	.71
**Substance 2 (N=100) Vaping dependence symptoms**	**Use of e-cigarettes with nicotine in past year**			**Vaped in past 30 days**			**Past year combustible cigarette use**		
	No (N=44)[a]	Yes (N=56)[a]	Test of Difference[b]	No (N=57)[a]	Yes (N=43)[a]	Test of Difference[b]	No (N=84)[a]	Yes (N=16)[a]	Test of Difference[b]
	N (%)	N (%)	P-Value	N (%)	N (%)	P-Value	N (%)	N (%)	P-Value
1 or more symptoms	0 (0.0)	6 (10.7)	.03	1 (1.8)	5 (11.6)	.04	3 (3.6)	3 (18.8)	.02*
2 or more symptoms	0 (0.0)	6 (10.7)	.03	1 (1.8)	5 (11.6)	.04	3 (3.6)	3 (18.8)	.02*
3 or more symptoms	0 (0.0)	4 (7.1)	.07	1 (1.8)	3 (7.0)	.19	1 (1.2)	3 (18.8)	.01*
**Substance 3 (N=118) Vaping dependence symptoms**	**Use of e-cigarettes with nicotine in past year**			**Vaped in past 30 days**			**Past year combustible cigarette use**		
	No (N=45)[a]	Yes (N=73)[a]	Test of Difference[b]	No (N=54)[a]	Yes (N=64)[a]	Test of Difference[b]	No (N=84)[a]	Yes (N=34)[a]	Test of Difference[b]
	N (%)	N (%)	P-Value	N (%)	N (%)	P-Value	N (%)	N (%)	P-Value
1 or more symptoms	5 (11.1)	11 (15.1)	.54	4 (7.4)	12 (18.8)	.07	12 (14.3)	4 (11.8)	.72
2 or more symptoms	2 (4.4)	8 (11.0)	.22	3 (5.6)	7 (10.9)	.30	6 (7.1)	4 (11.8)	.41
3 or more symptoms	2 (4.4)	4 (5.5)	.80	3 (5.6)	3 (4.7)	.83	4 (4.8)	2 (5.9)	.80
**Substance 4 (N=146) Vaping dependence symptoms**	**Use of e-cigarettes with nicotine in past year**			**Vaped in past 30 days**			**Past year combustible cigarette use**		
	No (N=37)[a]	Yes (N=109)[a]	Test of Difference[b]	No (N=53)[a]	Yes (N=93)[a]	Test of Difference[b]	No (N=60)[a]	Yes (N=86)[a]	Test of Difference[b]
	N (%)	N (%)	P-Value	N (%)	N (%)	P-Value	N (%)	N (%)	P-Value
1 or more symptoms	3 (8.1)	21 (19.3)	.11	3 (5.7)	21 (22.6)	.008*	8 (13.3)	16 (18.6)	.40
2 or more symptoms	0 (0.0)	14 (12.8)	.02*	1 (1.9)	13 (14.0)	.02*	3 (5.0)	11 (12.8)	.12
3 or more symptoms	0 (0.0)	10 (9.2)	.06	1 (1.9)	9 (9.7)	.07	1 (1.7)	9 (10.5)	.04

[a]Available data Ns for the respective variable and denominator for percentage. Total analytic sample N = 441 (Past-year e-cigarette users with vaping dependence data at baseline and past 6-month e-cigarette use data at 6-month follow-up; missingness N = 3).
[b]Test of group differences from χ^2 tests.
*Statistically significant after Benjamini-Hochberg corrections for multiple testing to control false discovery rate at .05 (based on two-tailed corrected P-value).

© 2020 Vogel EA et al. *JAMA Network Open.*

eTable 9. Interaction of Sex as a Moderator in Associations Between Electronic Cigarette Dependence Symptoms and Subsequent Vaping at 6-Month Follow-up

**E-cigarette use outcome at follow-up**		**Association of baseline e-cigarette dependence status × gender interaction term with outcome at follow-up[a]**		
		**Unadjusted[b]**		**Adjusted[c]**
		**P-Value**		**P-Value**
Any vaping over follow-up		.91		.55
Past 30-day number of nicotine vaping days		.08		.11
Past 30-day number of nicotine vaping sessions		.12		.16
Past 30-day puffs per nicotine vaping sessions		.27		.63

[a]Regression models of association between the interaction term of the hooked on nicotine checklist for e-cigarette dependence ($\geq$1 vs. 0 symptoms) and gender and respective outcome with a complex design accounting for clustering of data within schools.
[b]Unadjusted models include baseline status on the respective outcome as a covariate.
[c]Adjusted models include respective baseline outcome covariates and a propensity score for vaping dependence status derived from potentially confounding variables (i.e., youth gender, age, race/ethnicity, parental education level, e-cigarette use onset age, past year combustible cigarette smoking, past 30-day number of days smoked cigarettes, past 30-day number of cigarette smoked per day, ever use of cigar, hookah, or smokeless tobacco, ever alcohol use, ever smoked cannabis, ever vaped cannabis, ever other drug use, major depressive disorder, generalized anxiety disorder, panic disorder, social phobia, obsessive compulsive disorder, mania, ADHD, and conduct problems).

© 2020 Vogel EA et al. *JAMA Network Open.*

eTable 10. Interaction of Baseline Number of Nonnicotine Substances Used as a Moderator in Associations Between Electronic Cigarette Dependence Symptoms and Subsequent Vaping At 6-Month Follow-up

**E-cigarette use outcome at follow-up**		**Association of baseline e-cigarette dependence status × number of substance used interaction term with outcome at follow-up[b]**		
		**Unadjusted[c]**		**Adjusted[d]**
		**P-Value**		**P-Value**
Any vaping over follow-up		.17		.36
Past 30-day number of nicotine vaping days		.09		.12
Past 30-day number of nicotine vaping sessions		.33		.47
Past 30-day puffs per nicotine vaping sessions		.54		.70

[a]Non-nicotine substances include alcohol, cannabis, inhalants, cocaine, methamphetamine, LSD, ecstasy, heroin, salvia, prescription painkillers, tranquilizers/sedatives, diet pills, prescription stimulants, and bath salts.
[b]Regression models of association between the interaction term of the hooked on nicotine checklist for e-cigarette dependence (≥1 vs. 0 symptoms) and baseline number of substances used and respective outcome with a complex design accounting for clustering of data within schools.
[c]Unadjusted models include baseline status on the respective outcome as a covariate.
[d]Adjusted models include respective baseline outcome covariates and a propensity score for vaping dependence status derived from potentially confounding variables (i.e., youth gender, age, race/ethnicity, parental education level, e-cigarette use onset age, past year combustible cigarette smoking, past 30-day number of days smoked cigarettes, past 30-day number of cigarette smoked per day, ever use of cigar, hookah, or smokeless tobacco, ever alcohol use, ever smoked cannabis, ever vaped cannabis, ever other drug use, major depressive disorder, generalized anxiety disorder, panic disorder, social phobia, obsessive compulsive disorder, mania, ADHD, and conduct problems).

© 2020 Vogel EA et al. *JAMA Network Open.*

eTable 11. Past 30-Day Electronic Cigarette and Combustible Cigarette Use Patterns by Past 30-Day Number of Nicotine Vaping Days at Each Assessment

**Past 30-day number of nicotine vaping days**	**Past 30-day e-cigarette use**		**Past 30-day combustible cigarette use**	
	**Past 30-day number of nicotine vaping sessions**	**Past 30-day puffs per nicotine vaping sessions**	**Past 30-day number of days smoked cigarettes**	**Past 30-day number of cigarette smoked per day**
	***M* (*SD*)[c]**	***M* (*SD*)[c]**	***M* (*SD*)[c]**	***M* (*SD*)[c]**
**Baseline[a]**				
0 days (N=348)	0.39 (2.05)[4]	0.35 (1.61)[3]	0.58 (2.99)[3]	0.19 (0.89)[2]
1-2 days (N=45)	2.29 (4.05)[3]	3.47 (5.30)[2]	0.64 (1.20)[3]	0.40 (0.89)[2]
3-5 days (N=22)	4.85 (6.16)[2]	3.57 (2.48)[2]	3.00 (8.77)[2]	0.50 (1.19)[2]
6 or more days (N=26)	13.46 (7.30)[1]	10.81 (7.14)[1]	8.73 (10.85)[1]	4.80 (7.21)[1]
**6-month follow-up[b]**				
0 days (N=327)	0.18 (1.26)[4]	0.17 (1.05)[4]	0.75 (3.67)[3]	0.22 (1.19)[3]
1-2 days (N=40)	1.75 (2.68)[3]	2.18 (2.18)[3]	3.60 (6.85)[2]	1.03 (1.80)[2]
3-5 days (N=27)	4.00 (4.91)[2]	5.35 (5.31)[2]	2.52 (4.88)[2,3]	1.07 (1.64)[2]
6 or more days (N=46)	11.45 (8.23)[1]	6.39 (5.93)[1]	6.50 (10.33)[1]	2.11 (3.77)[1]

Abbreviations: N = Sample size; *M* = Mean; *SD* = Standard Deviation.
[a]Past 30-day e-cigarette and combustible cigarette use patterns by past 30-day number of nicotine vaping days at baseline (Available *N*s = 436 - 441).
[b]Past 30-day e-cigarette and combustible cigarette use patterns by past 30-day number of nicotine vaping days at 6-month follow-up (Available *N*s = 436 - 440).
[c]Calculated using one-way Analysis of Variance (ANOVA). Groups not sharing superscript numerals are significantly different in ANOVA Least Significant Difference. All F-statistics of ANOVA were significant (P-values based on two-tailed test < .001).

© 2020 Vogel EA et al. *JAMA Network Open.*

eTable 12. Interaction Effects of Electronic Cigarette Dependence Status and Other Drug Use at Baseline on Attention-Deficit/Hyperactivity Disorder Symptom Level at Follow-up

Baseline predictors	Associations of baseline e-cigarette dependence status and other substance use with ADHD symptom level at follow-up[a]			
	Unadjusted[b]		Adjusted[c]	
	b (95%CI)	p	b (95%CI)	p
**Main effects**				
e-cigarette dependence status[d]	0.38 (0.10, 0.66)	.008*	0.32 (0.07, 0.57)	.01*
Other substance use[e]	0.05 (-0.004, 0.11)	.06	0.04 (-0.01, 0.12)	.10
**Interaction effects[f]**				
e-cigarette dependence status[d] × Other substance use[e]	0.37 (0.08, 0.45)	.005*	0.22 (0.03, 0.41)	.02*

Abbreviations: b = unstandardized regression coefficient. 95%CI = 95% confidence interval.
[a]Generalized linear regression modeling was used with a complex design accounting for clustering of data within schools. Continuous ADHD level outcome was standardized.
[b]Regression models adjusting for baseline predictors and ADHD level.
[c]Regression models adjusting for baseline predictors, ADHD level, youth gender, age, race/ethnicity, parental education level, e-cigarette use onset age, past 30-day number of nicotine vaping days, past 30-day number of nicotine vaping sessions, past 30-day puffs per nicotine vaping sessions, past 30-day number of days smoked cigarettes, past 30-day number of cigarette smoked per day, other tobacco product use, major depressive disorder, generalized anxiety disorder, panic disorder, social phobia, obsessive compulsive disorder, mania, and conduct problems.
[d]Reporting $\geq$1 vs. 0 symptoms on the hooked on nicotine checklist for e-cigarette dependence.
[e]Other substance use was coded by sum of ever use of alcohol, combustible cannabis, vaped cannabis, and other drugs (Response range = 0 - 4).
[f]Interaction term was added in a subsequent model.
*Statistically significant after Benjamini-Hochberg corrections for multiple testing to control false discovery rate at .05 (based on two-tailed corrected P-value).

© 2020 Vogel EA et al. *JAMA Network Open.*

eTable 13. Prospective Associations of Baseline Electronic Cigarette Dependence Status With Combustible Cigarette Use Outcomes at Follow-up

Combustible cigarette use outcome at follow-up	N (%) / *M* (*SD*) of outcome at follow-up, by baseline e-cigarette dependence status[a]			Association of baseline e-cigarette dependence status[a] with outcome at follow-up[b]				
				Unadjusted			Adjusted[c]	
	Negative (N=392)	Positive (N=52)		OR (95%CI) / RR (95%CI)	P-Value		OR (95%CI) / RR (95%CI)	P-Value
Past 6-month cigarette use prevalence, N (%)	91 (23.3)	25 (48.1)		2.79 (1.39, 5.61)[f]	.009*		2.17 (0.99, 4.75)[f]	.05
Past 30-day number of days smoked cigarettes, *M* (*SD*)[d]	1.63 (5.46)	2.87 (6.52)		2.53 (1.74, 3.68)[g]	<.001*		2.33 (1.56, 3.52)[g]	<.001*
Past 30-day number of cigarettes smoked per day, *M* (*SD*)[e]	0.45 (1.49)	1.37 (3.38)		3.82 (2.43, 6.01)[g]	<.001*		3.03 (1.82, 5.04)[g]	<.001*

Abbreviations: OR = Odds ratio. RR = Rate ratio. 95%CI = 95% confidence interval.
[a]Reporting $\geq$1 vs. 0 symptoms on the hooked on nicotine checklist for e-cigarette dependence.
[b]Regression models of association between e-cigarette dependence status and respective outcome at follow-up with baseline status on the respective outcome as a covariate using a complex design accounting for clustering of data within schools.
[c]Additional adjusted for a propensity score for vaping dependence status derived youth gender, age, race/ethnicity, parental education level, combustible cigarette use onset age, past 6-month e-cigarette use, past 30-day number of nicotine vaping days, past 30-day number of nicotine vaping sessions, past 30-day puffs per nicotine vaping sessions, ever use of cigar, hookah, or smokeless tobacco, ever alcohol use, ever smoked cannabis, ever vaped cannabis, ever other drug use, major depressive disorder, generalized anxiety disorder, panic disorder, social phobia, obsessive compulsive disorder, mania, ADHD, and conduct problems (see eMethods for details on measures and propensity score calculation).
[d]Response range = 0 – 30.
[e]Response range: 0 – 20.
[f]Odds Ratio from logistic regression model for binary outcomes.
[g]Rate ratio from negative binomial regression model for count outcomes.
*Statistically significant after Benjamini-Hochberg corrections for multiple testing to control false discovery rate at .05 (based on two-tailed corrected P-value).

© 2020 Vogel EA et al. *JAMA Network Open.*

eReferences.

1. Miech R, Patrick ME, O'Malley PM, Johnston LD. What are kids vaping? Results from a national survey of US adolescents. *Tob Control*. 2016;26(4):386-391. doi:10.1136/tobaccocontrol-2016-053014
2. Cho J, Goldenson NI, Stone MD et al. Characterizing polytobacco use trajectories and Their associations with substance use and mental health across mid-adolescence. *Nicotine Tob Res*. 2018;20(suppl_1):S31-S38. doi:10.1093/ntr/ntx270
3. Goldenson NI, Leventhal AM, Stone MD, McConnell RS, Barrington-Trimis JL. Associations of electronic cigarette nicotine concentration with subsequent cigarette smoking and vaping levels in adolescents. *JAMA Pediatr*. 2017;171(12):1192-1199.
4. Chorpita BF, Yim L, Moffitt C, Umemoto LA, Francis SE. Assessment of symptoms of DSM-IV anxiety and depression in children: a revised child anxiety and depression scale. *Behav Res Ther*. 2000;38(8):835-855. doi:10.1016/s0005-7967(99)00130-8
5. American Psychiatric Association. Diagnostic and Statistical Manual of Mental Disorders. 4th ed, Text Revision.Washington, DC: American Psychiatric Publishing; 2004.
6. Hirschfeld RM, Williams JB, Spitzer RL et al. Development and validation of a screening instrument for bipolar spectrum disorder: The Mood Disorder Questionnaire. *Am J Psychiatry*. 2000;157(11):1873-1875. doi:10.1176/appi.ajp.157.11.1873
7. Barkley RA. Attention-Deficit Hyperactivity Disorder: A Clinical Workbook. 2nd ed. New York, NY: *Guilford Press*; 1998.
8. Thompson MP, Ho C, Kingree JB. Prospective associations between delinquency and suicidal behaviors in a nationally representative sample. *J Adolesc Health*. 2007;40:232-237.
9. Brookhart MA, Schneeweiss S, Rothman KJ, Glynn RJ, Avorn J, Stürmer T. Variable selection for propensity score models. *Am J Epidemiol*. 2006;163(12):1149-1156. doi:10.1093/aje/kwj149
10. Rubin DB, Thomas N. Matching using estimated propensity scores: Relating theory to practice. *Biometrics*. 1996;52(1):249-264. doi:10.2307/2533160

© 2020 Vogel EA et al. *JAMA Network Open.*

11. Austin PC. Some methods of propensity-score matching had superior performance to others: Results of an empirical investigation and Monte Carlo simulations. *Biom J*. 2009;51(1):171-184. doi:10.1002/bimj.200810488

© 2020 Vogel EA et al. *JAMA Network Open.*
